# Assessment of *Culex pipiens* bioforms in the world’s southernmost distribution limit

**DOI:** 10.1590/0074-02760190390

**Published:** 2020-02-07

**Authors:** María Victoria Cardo, Alejandra Rubio, Darío Vezzani, Aníbal Eduardo Carbajo

**Affiliations:** 1 Universidad Nacional de San Martin-Consejo Nacional de Investigaciones Científicas y Técnicas, Instituto de Investigación e Ingeniería Ambiental, Ecología de Enfermedades Transmitidas por Vectores (2eTV), San Martín, Provincia de Buenos Aires, Argentina; 2 Consejo Nacional de Investigaciones Científicas y Técnicas, Buenos Aires, Argentina; 3 Universidad Nacional del Centro de la Provincia de Buenos Aires, Facultad de Ciencias Exactas, Instituto Multidisciplinario sobre Ecosistemas y Desarrollo Sustentable, Tandil, Provincia de Buenos Aires, Argentina

**Keywords:** aboveground, Culex pipiens molestus, distribution, Patagonia, St. Louis encephalitis, West Nile virus

## Abstract

The mosquito *Culex pipiens s.s.* L. occurs as two bioforms that differ in physiology and behaviour affecting virus transmission cycles. To assess the occurrence of *Cx. pipiens* bioforms in the southernmost limit of its distribution, specimens were collected aboveground in southern Buenos Aires Province and east Patagonia, Argentina. Ten larvae and 25 adults were individually processed and identified by polymerase chain reaction (PCR) amplification of Ace-2 and CQ11 loci. *Culex quinquefasciatus* Say (one larva, two adults), *Cx. pipiens* f. *molestus* (one larva, one adult) and one adult of hybrid origin were identified in Buenos Aires Province; only *Cx. pipiens* f. *molestus* was recorded in Patagonia (eight larvae, 21 adults). The potential absence of bioform *pipiens* and its implications in arbovirus enzootic cycles is discussed.

The mosquitoes grouped in the *Culex pipiens* L. complex (Diptera: Culicidae) are cosmopolitan vectors of diseases that affect humans, companion and productive animals, and wildlife.^1^ Among them, West Nile virus (WNV) is the most widespread cause of arboviral neurological disease in the world; it is responsible for large human outbreaks in Europe and North America, as well as ongoing transmission in the Middle East, Africa and Asia.^2^ In Argentina, WNV has been isolated from horses^3^ and antibodies detected in free-ranging birds,^4^ with few sporadic human cases reported.^5^ St. Louis encephalitis virus (SLEV) is also broadly distributed in the Western Hemisphere, particularly in temperate regions. Although less than 1% of human infections develop symptoms, the disease is severe for other mammals as dead-end hosts.^6^ In Argentina, human cases of SLEV were reported sporadically until an unprecedented outbreak in 2005, followed by case clusters in 2006, 2010, 2011, 2013 and 2019.^7^ ^,^ ^8^

In the Americas, the species complex consists of *Culex quinquefasciatus* say from tropical to temperate areas and the nominal species with two bioforms from temperate to cold regions. The bioform *pipiens* has been described as ornithophilic, heterodynamic, anautogenous and eurygamous, whereas *molestus* is mammophilic, homodynamic, autogenous and stenogamous.^9^ These ecophysiological differences have implications in disease transmission, increased by the fact that hybrids show opportunistic feeding behaviour and may therefore serve as bridge vectors for virus transmission from infected birds to humans or other dead-end hosts.^10^

The aim of this work was to address for the first time the presence of *Cx. pipiens* bioforms in the world’s southern extreme of its distribution. Sampling was conducted from southern Buenos Aires Province to east Patagonia region along the Atlantic coast (Figure), under the hypothesis that bioform *molestus* is present in the northern extreme of the study area and is gradually replaced by bioform *pipiens* in the southern end. Buenos Aires Province is characterised by cumulative precipitation values between 500 and 1,100 mm west-east and a mean annual temperature gradient ranging from 13.4ºC southeast to 17.9ºC northwest;^11^ isotherms present a non-flat pattern as the result of the joint interaction of topography and oceanic influence. Argentinean Patagonia extends over the southern cone of South America, limited by the Colorado River to the north, the Atlantic Ocean to the east and the Andean cordillera to the west (Figure). It includes a wide variety of soils, climatic conditions and vegetation formations; mean annual temperatures range from 16ºC in the northwest to 3ºC in the south.^11^ The eastern portion of the territory is drier than the west (< 250 mm per year), as the zonal atmospheric flow and transport of humid air from the Pacific Ocean are blocked by the Andes Cordillera. The arid steppe is intersected by four main rivers, which margins concentrate most human settlements. All specimens of *Cx. pipiens* previously collected throughout Patagonia have been identified by morphological or enzymatic techniques; therefore, no bioform differentiation is available.^12^ ^,^ ^13^

Specimens of *Cx. pipiens s.l.* were collected during March 2018 and January 2019 along and in the vicinities of the key communication route between southern Buenos Aires and east Patagonia, covering over 1700 km from 36.36 to 47.18ºS (Figure). Collections were performed in cemeteries, tire repair shops, river margins, and public toilets of fuel stations or other facilities. Adult mosquitoes were caught with manual and battery-powered hand aspirators whereas immatures were collected by dipping in discarded vehicle tires, uncovered water tanks and flower vases. All specimens were preserved at -16ºC in a portable car freezer until arrival to the laboratory. There, DNA from individual specimens (either larvae or adults) was extracted with one of the following methods: approximately 50% were individually ground with sterilised mortar and pestle and genomic DNA was extracted using the EasyPure Genomic DNA extraction kit (Transgen Biotech), whereas DNA from the other 50% was extracted with 4 M ammonium hydroxide following Vogels et al.^14^ The second inexpensive method was used for sampling locations with many specimens, whereas silica extraction columns were reserved for scarce samples (one or two individuals at a given location).

Specimen identification followed the polymerase chain reaction (PCR) protocols by Smith & Fonseca^15^ and Bahnck & Fonseca^16^ for the amplification of the second intron of the Ace-2 nuclear gene and the 5′ flanking region of microsatellite locus CQ11, respectively. The first protocol amplifies a 610-bp band for *Cx. pipiens* and a 274-bp band for *Cx. quinquefasciatus* [Supplementary data **(Figure)**], whereas the simultaneous presence of both bands is indicative of hybrid signatures. The second distinguishes between both forms of *Cx. pipiens* amplifying a 250-bp band for *molestus* and a 190-200-bp band for *pipiens*; again, the presence of both bands indicates hybrid signatures. As a result of cross-reactivity of primers, *Cx. quinquefasciatus* amplifies a PCR product similar in size to that of *Cx. pipiens* f. *molestus* in the CQ11protocol [Supplementary data **(Figure)**]; therefore, the combined use of both is mandatory in sympatric areas. A negative (distilled water) control and positive controls from *Cx. pipiens* f. *pipiens* (Southern France), *Cx. pipiens* f. *molestus* (England) and *Cx. quinquefasciatus* (Indonesia) were included in all runs. A 5 μL aliquot of each amplified product mixed with 1 μL of loading buffer 6x was electrophoresed in a 2% agarose gel containing ethidium bromide (0.5 μg/mL) and 0.5X TBE buffer. Bands were visualised under a gel UV transilluminator. DNA ladders of 50 and 100 bp precision were run in parallel to allow size estimation of observed bands. Amplified PCR product of the southernmost collected specimen was purified with DNA PuriPrep-GP kit (INBIO Highway) and sequenced in an ABI 3130xl Genetic Analyzer (Applied Biosystems) by a third-party provider. Sequences were edited using ApE v2.0.55 and compared to known sequences by a BLAST search comparison with the GenBank DNA database (www.ncbi.nlm.nih.gov/blast/Blast.cgi).

We collected specimens of *Cx. pipiens s.l.* from Tapalqué (Buenos Aires Province, 36.36ºS) to Comodoro Rivadavia (Chubut Province, 45.87ºS) in cemeteries (two positive / four inspected), tire repair shops (5/10), river margins (2/5) and public toilets (8/32) (Table). Only adults or larvae were collected at any given positive site with the exception of a cemetery (site 28), in which immatures were collected in a flower vase and one female was aspirated from the vegetation surrounding the same grave. Adult collections were performed mainly in public toilets of fuel stations (sites 1, 2, 11, 21, 22, 24, 25 and 33) or cemeteries (site 41); in one tire repair shop, all tires were dry but adults were resting inside its cavities (site 6). A large number of adult specimens were recorded in humid environments associated with river margins; at a camping site (site 12), plenty of adults were collected beneath grill spots and inside rubber dustbins, whereas at a canal near the river (site 35) adults were found resting under a tree near the ground. Larval collections were performed in vehicle tires at sites 4, 10, 13 and 18 (Table). South of Comodoro Rivadavia (location P in Figure), we inspected four public toilets, one tire repair shop, one cemetery and one river margin but no adults were found and all potential larval habitats were dry (details in Table footnote).

**TABLE:** Members of the *Culex pipiens* complex by sampling site, date and land use

Location	Site	Locality (Province)	Date	Land use	Larvae	Adults	ID (n)
A	1	Tapalqué (BA)	31-Mar-2018	Public toilet		+	P (1)
A	2	Tapalqué (BA)	31-Mar-2018	Public toilet		+	H (1)
B	4	Coronel Pringles (BA)	25-Jan-2019	Tire repair shop	++		P (1)
C	6	Bahía Blanca (BA)	25-Jan-2019	Tire repair shop		++	Q (2)
D	10	Médanos (BA)	25-Jan-2019	Tire repair shop	+		Q (1)
E	11	La Adela (LP)^*a*^	25-Jan-2019	Public toilet		+	P (1)
E	12	Río Colorado (RN)	25-Jan-2019	Camping at river margin		+++	P (2)
E	13	Río Colorado (RN)	25-Jan-2019	Tire repair shop	++		P (2)
G	18	San Antonio Oeste (RN)	24-Jan-2019	Tire repair shop	++		P (2)
H	21	Sierra Grande (RN)	31-Mar-2018	Public toilet		+	P (1)
H	22	Sierra Grande (RN)	24-Jan-2019	Public toilet		+	P (2)
I	24	Punta Norte (CH)	30-Mar-2018	Public toilet		+	P (1)
J	25	Puerto Pirámides (CH)	30-Mar-2018	Public toilet		+	P (1)
K	28	Puerto Madryn (CH)	24-Jan-2019	Cemetery	++	+	P (7)
L	33	Trelew (CH)	24-Jan-2019	Public toilet		+	P (1)
M	35	Gaiman (CH)	24-Jan-2019	River margin		+++	P (8)
P	41	Comodoro Rivadavia (CH)^*b*^	23-Jan-2019	Cemetery		+	P (1)

Location letters correspond to those in Figure. In column ID (for identification), P, Q and H indicate *Cx. pipiens* f. *molestus*, *Cx. quinquefasciatus* and the hybrid between the two, respectively; the number of identified specimens is reported between brackets (n). Province code: BA: Buenos Aires; LP: La Pampa; RN: Rio Negro; CH: Chubut; SC: Santa Cruz. Relative abundance of larvae and adults: + low, ++ intermediate, +++ high. *a*: La Adela is next to Río Colorado just across the Colorado River; *b*: 1 female was sequenced. The number of negative sites in each location were: Location B: one public toilet; C: two public toilets; D: two public toilets, one tire repair shop; E: one public toilet; F: two public toilets, one river margin; G: two public toilets; H: one tire repair shop; K: three public toilets; L: two public toilets, one river margin; M: one cemetery; N: one public toilet; O: two public toilets; P: three public toilets, two tire repair shops; Q: two public toilets, one cemetery; R: two public toilets, one tire repair shop; S: one river margin.

**Figure:**
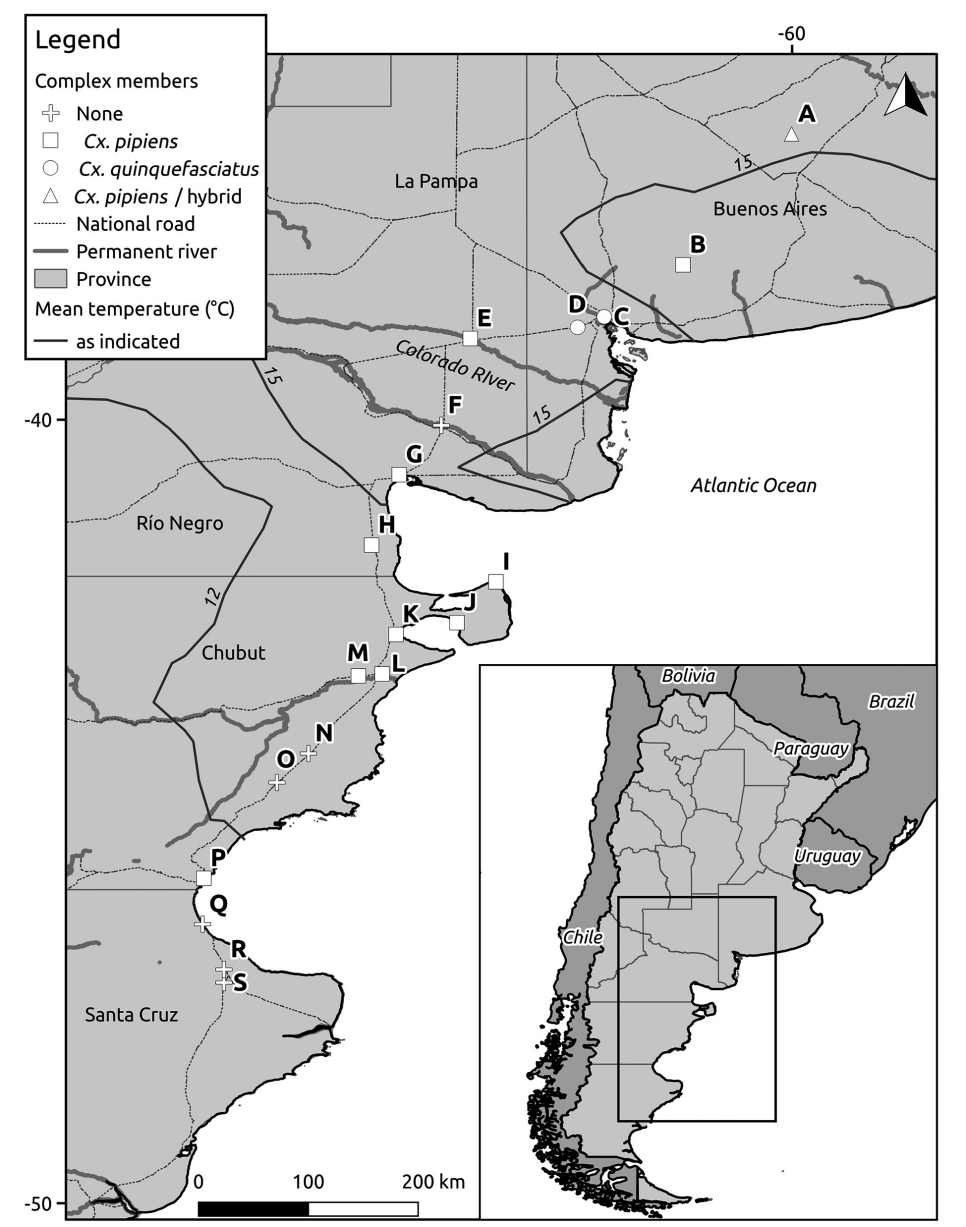
Occurrence of the members of the *Culex pipiens* complex in southeast Argentina. Letters indicate sampling locations, see Table for details.

A total of 10 larvae and 25 adults were individually processed; all DNA extractions using both methods resulted in successful PCR identifications. Specimens were identified either as *Cx. quinquefasciatus*, *Cx. pipiens* f. *molestus* or hybrids between the two, remarking the absence of *Cx. pipiens* f. *pipiens* [Supplementary data (Figure)]. Both *Cx. quinquefasciatus* (one larva, one female, one male), *Cx. pipiens* f. *molestus* (one larva, one female) and a hybrid *Cx. quinquefasciatus*/*Cx. pipiens* f. *molestus* (one female) were collected in Buenos Aires Province, whereas *Cx. pipiens* f. *molestus* was the only member of the complex recorded throughout east Patagonia (eight larvae, 15 females, six males). Top blast matches of the sequence of the CQ11 amplified PCR product of the female collected at Comodoro Rivadavia were *Cx. pipiens* f. *molestus* (100% identity and query cover with reference specimen DQ470149^16^).

The results reported herein represent first-to-date molecular identifications of *Cx. pipiens* bioforms in its world’s southernmost distribution. Although in small numbers, we collected both larvae and adults of the complex in different land uses, and all specimens of *Cx. pipiens s.s.* were consistently identified as form *molestus*. Previous studies in Buenos Aires Province identified specimens from La Plata City (34.87ºS, 57.90ºW) as form *molestus* by a full microsatellite analysis,^17^ whereas the individuals collected in 13 cemeteries throughout the province were also identified as *molestus* using the CQ11 locus.^18^ Although no previous attempts to identify *Cx. pipiens* bioforms had been made in Patagonia, Mitchell et al.^19^ reported a high degree of autogeny and proof of anthropophily in a colony established from mosquitoes collected at 43ºS. This constitutes a worthy of note precedent of the occurrence of form *molestus* in the distribution fringe of *Cx. pipiens*, and the potential absence of form *pipiens* territory wide. Although the *molestus* form was originally considered to be limited to belowground and confined breeding sites, during the past decade it has been reported to occur naturally in open and aboveground habitats, even at high latitudes in the Northern Hemisphere with more extreme climatic conditions than those recorded in Patagonia (e.g.^14^). Our current finding in Comodoro Rivadavia is the southernmost record of *Cx. pipiens* f. *molestus* in South America.

Argentinean members of the *Cx. pipiens* complex were reported to be competent but only moderately efficient vectors and less susceptible to WNV than comparable U.S. mosquito strains.^17^ Although no attempt to isolate WNV or SLEV from wild avifauna or mosquitoes have been conducted in Patagonia, these arboviruses have been reported throughout North America at latitudes similar or even higher than the ones in to the southern counterpart.^2^ ^,^ ^7^ If the bioform *pipiens* is actually absent, other *Culex* species could be responsible for the maintenance of arbovirus enzootic cycles. Alternatively, in Patagonia *Cx. pipiens* f. *molestus* could show opportunistic behaviour and feed on birds. In North America, at the beginning of the season *Cx. pipiens* has been reported to feed on birds (particularly the American robin *Turdus migratorius*), whereas after bird migration it switches to human feeding, therefore acting as an excellent bridge vector.^20^ No host preference studies of the bioforms have been yet conducted in South America, highlighting an information gap to be fulfilled by future studies together with the aim of drawing a more complete distribution map of the bioforms in southern Argentina.

Our biotyping method assigns specimens into one of three possible discrete outcomes (*molestus*, *pipiens* or their hybrids) based on a single locus. Although this assay is considered a reliable diagnostic method, we acknowledge that the genetic structure of the *Cx. pipiens* bioforms is far more complex. To shed light on this matter and gain insight in the evolutionary relationships of the South American strain, selected specimens were recently included in a whole genome sequencing project currently under development, along with other specimens from all around the global range of the species.
