# Supplementary material for: Assessment of Culex pipiens bioforms in the world’s southernmost distribution limit
Source: Mem Inst Oswaldo Cruz. 2020 Feb 7;115:e190390. doi: 10.1590/0074-02760190390 (PMC7012581; doi:10.1590/0074-02760190390)
Supplement: Supplementary file 1 [file 1678-8060-mioc-115-e190390-s.pdf]

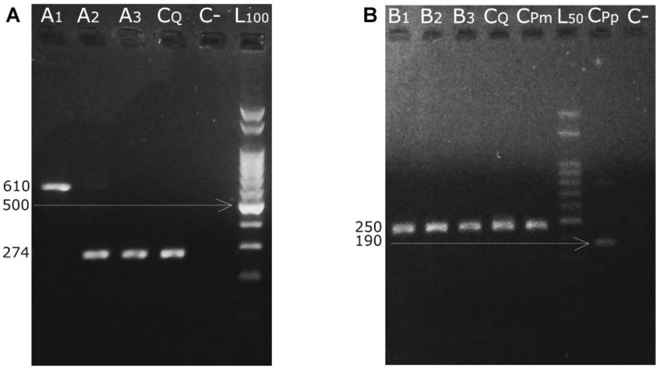

Gel images of electrophoretic runs of polymerase chain reaction (PCR) products from Ace-2 locus (A) and CQ11 locus (B). Lanes A1 and B1 represent one female of site 41 (*Culex pipiens* f. *molestus*); lanes A2 and B2, one female from site 6 (*Cx. quinquefasciatus*); and lanes A3 and B3, one larva from site 10 (*Cx. quinquefasciatus*). CQ, CPm and CPp indicate positive controls for *Cx. quinquefasciatus*, *Cx. pipiens* f. *molestus* and *Cx. pipiens* f. *pipiens*, respectively. C- is the negative control (distilled water). L_{100} and L_{50} are ladders of 100 and 50 bp precision, respectively. Numbers at the left of each image indicate the estimated weight of the obtained bands (in bp).
